# Calcific Aortic Valve Stenosis: A Focal Disease in Older and Complex Patients—What Could Be the Best Time for an Appropriate Interventional Treatment?

**DOI:** 10.3390/jcm14155560

**Published:** 2025-08-07

**Authors:** Annamaria Mazzone, Augusto Esposito, Ilenia Foffa, Sergio Berti

**Affiliations:** 1 Fondazione Toscana Gabriele Monasterio Massa, 54100 Massa, Italy; ilenia.foffa@cnr.it (I.F.); berti@ftgm.it (S.B.); 2 Istituto di Fisiologia Clinica, Consiglio Nazionale delle Ricerche, 54100 Massa, Italy

**Keywords:** management of severe aortic stenosis, older patient, appropriate interventional treatment, biomarkers

## Abstract

Calcific aortic stenosis (CAS) is a newly emerging pandemic in elderly individuals due to the aging of the population in the world. Surgical Aortic Valve Replacement (SAVR) and Transcatheter Aortic Valve Replacement (TAVR) are the cornerstone of the management of severe aortic stenosis accompanied by one or more symptoms. Moreover, an appropriate interventional treatment of CAS, in elderly patients, is a very complex decision for heart teams, to avoid bad outcomes such as operative mortality, cardiovascular and all-cause death, hospitalization for heart failure, worsening of quality of life. In fact, CAS in the elderly is not only a focal valve disease, but a very complex clinical picture with different risk factors and etiologies, differing underlying pathophysiology, large phenotypic heterogeneity in a context of subjective biological, phenotypic and functional aging until frailty and disability. In this review, we analyzed separately and in a more integrated manner, the natural and prognostic histories of the progression of aortic stenosis, the phenotypes of myocardial damage and heart failure, within the metrics and aging trajectory. The aim is to suggest, during the clinical timing of valve disease, the best interval time for an appropriate and effective interventional treatment in each older patient, beyond subjective symptoms by integration of clinical, geriatric, chemical, and advanced imaging biomarkers.

## 1. Introduction

Calcific aortic stenosis (CAS), due to the aging of the population in the Western world, represents a newly emerging pandemic in elderly individuals. The prevalence of CAS in adults over 75 years, is 10–15%, but is expected to more than double by 2040, having a major impact on health care systems [1].

Surgical Aortic Valve Replacement (SAVR) and Transcatheter Aortic Valve Replacement (TAVR) are the cornerstone of the management of severe aortic stenosis (AS) accompanied by one or more symptoms (pain, syncope or near syncope), resuscitate sudden death, shortness of breath, fatigue, effort intolerance, or left ventricular dysfunction. Aortic valve replacement is advised due to the well-known poor prognosis (50% mortality rate within three years) in symptomatic cases that do not undergo surgery, along with the generally favorable surgical results and comparatively low rates of perioperative mortality and complications, even in octogenarians [2,3]. In recent decades, following the development of TAVR, the treatment of severe aortic stenosis has been revolutionized [3]. Moreover, despite improvements in outcomes, elderly individuals with multiple comorbidities and geriatric syndromes may experience detrimental effects on mortality and quality of life, highlighting the cumulative vulnerability that persists despite valve replacement, and the futility of interventional treatment [4]. The advancement of TAVR, which was first researched among elderly patients with prohibitive or high surgical risks for valve replacement, has identified that we can gradually enhance the stratification of these populations by incorporating the idea of frailty and other geriatric risks. This approach allows for a personalized diagnosis and a thorough management of aortic stenosis in older individuals, ultimately improving shared decision-making with patients and their families while aiming to optimize outcomes centered around the patient [4,5]. Physicians treating patients with aortic stenosis often encounter the challenge of deciding how to manage an elderly individual (>75 years old) who exhibits both physical and echocardiographic indicators of severe AS but reports feeling asymptomatic or has symptoms obscured by significant comorbidities that hinder their usual daily activities [4,6].

An appropriate interventional treatment of CAS, in elderly patients, is a very complex decision for heart teams, to avoid bad outcomes such as operative mortality, cardiovascular death and all-cause death, hospitalization for heart failure, and worsening of quality of life [3]. Heart team assessment must take into consideration the coexistence of multiple heterogeneous factors, as well as the structural and functional abnormalities of valves and heart, the degree of comorbidity and frailty and the final clinical effects of their mutual interactions in the older patient [3,4,5]. The approach to assessing risk in elderly patients with aortic stenosis is likely most effective through a thorough clinical evaluation of when to intervene, particularly for those with valvular disease that is more than mild, to ensure that timely treatment provides the greatest benefit [7,8]. Furthermore, randomized clinical trials on the effects of early intervention in patients with asymptomatic severe AS support a reconsideration of current practice patterns. A recent meta-analysis of four randomized controlled trials that compared clinical surveillance versus interventional management in asymptomatic patients with severe AS highlights that early aortic valve replacement (AVR) is associated with a significant lower risk for heart failure (HF) or unplanned cardiovascular hospitalizations, stroke and with a no significant differences in all-cause or cardiovascular (CV) mortality compared to clinical surveillance [9].

While recent literature data seem to clarify the need for interventional timing in patients with asymptomatic severe calcific aortic stenosis, there is still little data regarding the relationship among the progression of calcific aortic stenosis, the phenotypic heart failure and the biological/functional age in older patient, to detect the better relative time window for an appropriate interventional treatment that can improve the patient′s prognosis.

### Natural History and Outcomes of Calcific Aortic Stenosis in the Elderly

Calcific aortic stenosis, the third-most frequent cardiovascular disease after coronary artery disease and systemic arterial hypertension, is characterized by progressive fibro-calcific remodeling and thickening of the aortic valve leaflets that, over years, evolve to cause severe obstruction to cardiac outflow. Congenital abnormality as bicuspid valve and older age are powerful risk factors for calcific AS [3]. Aortic sclerosis, the preclinical phase of disease, is characterized by focal areas of valve calcification and leaflet thickening without significant cardiac blood flow obstruction (aortic jet velocity of <2.0 m/s) and may be present and asymptomatic for years to decades. A mild obstruction occurs in all patients as hemodynamic progression, typically over 5–10 years, with no associated symptoms or exercise limitation. Patients are generally asymptomatic unless they have other comorbidities that contribute to the emergence of symptoms [10]. Imaging modality (echocardiography, CT aortic valve, contrast CT angiography, PET/CT) and biomarker monitoring (BNP/NT-proBNP, cardiac troponin, inflammatory, metabolic and oxidative stress markers, lipid profile, lipoprotein(a), micro-RNA) reflect the complex pathophysiology of the processes. In fact, the pathophysiology of calcific AS is complicated and includes active leaflet calcification, chronic inflammation, lipoprotein oxidation and deposition, osteoblastic transformation of heart valve interstitial cells, and hereditary variables. An increased risk of CAS has been linked to the metabolic syndrome and an elevated lipoprotein(a) plasma level [3]. Although no medication has been found to be successful in slowing the progression of AS, a number of indicators, including ectonucleotidases, the renin-angiotensin system, receptor activator of NF-κB ligand, and lipoprotein (a), may be novel targets for treatment [3]. A hybrid approach that combines lifestyle changes, mechanical or ultrasonic decalcifying procedures, and some regularly used medications may slow the advancement of CAS. A higher probability of symptom development is linked to significant valve calcification, advanced age, and faster disease progression [11]. The progression of calcific AS results in a progressive reduction in the aortic valve area, which accelerates the flow. Patients with severe AS could have symptoms [12], and most adults with severe AS develop symptoms within a few years of the diagnosis and need more frequent clinical and Doppler-echocardiographic follow-up than those with mild or moderate forms of the disease because AS severity is a strong predictor of clinical outcome [12,13]. Valve replacement in asymptomatic patients includes some advantages such as prevention of left ventricular hypertrophy and diastolic dysfunction, but there are no studies that show that early surgery could prevent such changes in left ventricular structure and function with an acceptable risk–benefit ratio. The risk of sudden death due to CAS is substantial once symptoms develop; however, in the absence of symptoms, the risk appears to be minimal (<1%/yr), even in patients with severe disease [14]. Valve replacement is currently not recommended for prevention of sudden cardiac death in asymptomatic AS [7,8].

## 2. Calcific AS Is Not Only a Valve Disease: Phenotypes of Myocardial Damage and Heart Failure in the Natural History

Aortic stenosis is not only a disease of the aortic valve but also involves ventricles, and multimodality imaging can help to evaluate the left ventricular (LV) remodeling response and to improve risk stratification in patients with moderate AS [13]. In CAS natural history, during asymptomatic clinical time, the left ventricle responds to the increased pressure load with adaptive concentric wall hypertrophy that maintains wall stress and the left ventricular ejection fraction (LVEF). However, at this point, LV diastolic filling and Left Ventricular Longitudinal Shortening are already compromised. In more advanced stages of CAS, the inability of LV to counterbalance the pressure overload leads to a reduced LVEF, heart failure symptoms, and poor outcomes. Heart failure symptoms with either a preserved ejection fraction (HFpEF) or a reduced ejection fraction (HFrEF) can be seen in up to 30% of individuals with aortic stenosis [15]. Concomitant coronary heart disease is another important cause of HF in AS patients and has an important therapeutic and prognostic implications. Extra valvular risk parameters can inform aortic stenosis risk stratification. Therefore, moderate AS may have a prognosis comparable to severe aortic stenosis if there is cardiac injury or dysfunction [16].

Current guidelines recommend clinical surveillance for patients with moderate AS, and aortic valve replacement may be considered if there is an indication for coronary revascularization [7,8]. However, in patients with moderate AS, the potential benefit related to early AVR is unknown [16]. HF development in AS is multifactorial and its link to left ventricular dysfunction is a complex process. Delineating the determinants of HF admissions in AS is crucial for identifying individuals at high risk. Identifying the early signs of left ventricular decompensation, using surrogate markers may be the key, even before left ventricular function becomes impaired. By combining multimodality imaging techniques and biomarkers to assess cardiac damage and procedural factors that affect HF before and after, AVR can facilitate timely intervention, reduce the risk of HF progression and have an impact of future recommendations [15].

Because there are confounding factors such as symptoms, morbidities, and concomitant left ventricular dysfunction, in elderly patients, the treatment threshold remains undefined [17]. Current clinical decision paradigms may be drastically altered by evolving data based on imaging and biomarkers linked to unfavorable ventricular remodeling, hypertrophy, inflammation, or fibrosis [15]. The clinical course of heart failure is characterized by a trajectory with an early relatively stable phase of chronic disease also compensated by multiple therapies for cardiac dysfunction and comorbidities. Further, the continuum of HF is characterized by a progressive deterioration with bouts of (often unpredictable) acute or subacute decompensation from fluid overload, overdiuresis, non-adherence, infection or other intercurrent [18].

Decompensated acute HF (AHF) events and hospitalization, as complications of severe AS in older patients, were associated with an extremely dismal prognosis, with worse short- and long-term outcomes. In addition, in this setting of patients, early TAVR not significantly reduces all causes of mortality at 30 days [19].

Furthermore, Esposito et al. have recently highlighted that in elderly patients with severe AS and an ejection fraction equal to 50%, treated with either interventional or pharmacological therapy, a previous event of AHF with hospitalization is independently associated with advanced degree of frailty phenotype, and poor outcomes [20].

HF decompensation remains the leading cause of cardiac rehospitalization and a major predictor of mortality in patients with AS, before or after AVR [21].

*Main points*

- *Aortic stenosis is not only a disease of the aortic valve but also involves the ventricle. HF decompensation remains the leading cause of cardiac rehospitalization and a major predictor of mortality in patients with AS, before or after AVR.*
- *Moderate AS may have a prognosis comparable to severe aortic stenosis if there is cardiac injury or dysfunction.*
- *Multimodality imaging techniques and biomarkers to assess cardiac damage and procedural factors that affect HF before and after AVR can facilitate timely intervention, reduce the risk of HF progression and have an impact of future recommendations.*

## 3. Calcific AS in the Elderly Is Not Only a Cardiovascular Disease: The Weight of Frailty in the Trajectory of Aging

Elderly with severe CAS are heterogeneous and complex patients, including fit older subjects, but also patients with different levels of cognitive and functional impairment, multimorbidity, polypharmacy, sarcopenia, disability and frailty [4,5].

Frailty, a complex age-related clinical condition, is characterized by a decline in physiological capacity across several organ systems that increases the susceptibility to stressors. According to clinical data, 38.4% of older people (mean age 84.6 ± 4.4 years) with severe AS were also frail; in particular, the prevalence of frailty was 59.6% among patients aged ≥ 70 years with asymptomatic severe AS, while among patients aged ≥ 75 years, it was 49.3% and frailty was independently associated with mortality [22]. However, frailty is highly complicated in patients with AS, regardless of whether they are symptomatic or asymptomatic [22,23] and while there are adequate guidelines for treating AS in adults, there are many gaps in the management of elderly frail patients with AS.

Frailty develops as a continuum from being robust and independent to being at risk of disability and dependency, to being hospitalized, institutionalized or at risk of dying [24]. Younger robust individuals are more likely to recover quickly from an injury or illness that reduces their functional capacity. In the course of life, cumulative physiological decline across multiple body systems leads to episodic functional, psychological or cognitive decompensation. Progression of frailty status increases incident cardiovascular disease risks, while recovery of frailty status decreases incident CVD risks [25].

Pre-frailty is an intermediate state between frailty and non-frailty/robust that has higher risk of progressing to frailty but that is identified as a potentially reversible condition and as the ‘entry door’ into the process may represent a key stage that could offer new opportunities for early, targeted, individualized and tailored interventions and care in clinical geriatrics. The trajectory of the frailty syndrome can be modified via physical activity and nutritional interventions [4,26]. For the majority of diseases, aging is thought to be the main risk factor. Different racial, social, environmental, and lifestyle factors can contribute to systemic chronic inflammation, which can lead to a number of diseases, including cancer, diabetes mellitus, cardiovascular disease, chronic kidney disease, non-alcoholic fatty liver disease, autoimmune, and neurodegenerative disorders. However, there is a continuum of aging and age-related diseases with common pathophysiologic mechanisms [27].

In older adults, aortic valve stenosis, heart failure, comorbidities and frailty are strongly conditioned for the modality of expression, throughout life, by time and quality of aging.

In this regard, Ferrucci et al. [28] described three different metrics, biological, phenotypical and functional age, as mutually and longitudinally correlated but their trajectories as asynchronous.

Chronological aging refers to the time-dependent decline in physical and biologic functions that occurs at different rates among patients as the result of the interplay between genetics, biology, and environmental factors. Biological aging is defined as the rate at which an individual ages, referring to the individual’s variability in the age-associated declines [4]. Therefore, differentiating biological aging from chronological aging plays a crucial role in older patients’ selection and aids in more accurately stratifying patients who are candidates for TAVR/SAVR [29]. Biological aging (molecular and cellular aging) takes many years before it translates into the deterioration of physical/cognitive function (phenotypical age) and later functional aging only occurs when the resilience mechanisms of biological and phenotypic aging are exhausted; frailty and disability are powerful risk factors for multiple adverse health outcomes, such as hospitalization and mortality. Frailty and other measures of functional aging have been used to identify patients most likely to develop severe side effects after aggressive medical/surgical treatments.

New methods to measure the biological mechanisms of aging in humans may enable identification of individuals on a trajectory of accelerated aging early in the process, and subclinical diseases and thereby targeted for future interventions that globally affect the aging rate and delay frailty [29]. Therefore, “Gerotherapeutics” may became a new technological innovation in measuring the difference between chronological and biological aging [30].

Novel biomarkers need to be fully developed, validated, and included in large-cohort studies in addition to the major phenotypes of aging, including information on chronic diseases and risk factors, as well as sensitive measures of functional assessment

*Main points*

- *Calcific aortic stenosis (CAS) is the most common valvular heart disease in the elderly, and elderly with severe CAS are heterogeneous and complex patients.*
- *38.4% of older people with severe AS were also frail.*
- *In older adults, aortic valve stenosis, heart failure, comorbidities and frailty are strongly conditioned for the modality of expression, throughout life, by time and quality of aging.*
- *Differentiating biological aging from chronological aging plays a crucial role in older patients’ selection and aids in more accurately stratifying patients who are candidates for TAVR/SAVR.*

### What Could Be the Best Time for an Appropriate Interventional Treatment of Calcific Aortic Stenosis in the Elderly?

A multidimensional heart team assessment is necessary to avoid futility and potential harmful of AS interventional treatment. In particular, it is useful to establish the best timing for an effective interventional treatment of the CAS in relation to the trends of the progression of the valvular disease, myocardial damage and biological age of older patients. Performing a correct diagnosis in frail and aged population with AS is a vital problem that often leads to a timing delay in delivering interventional options.

**Figure 1:**
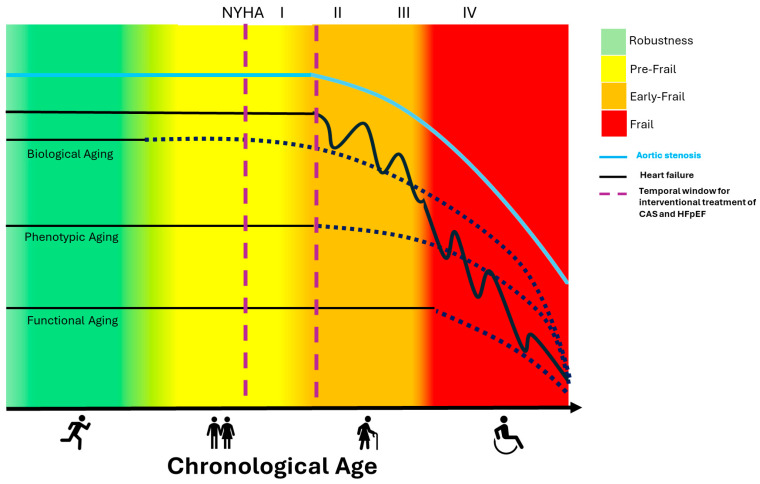
Trajectories of the natural histories of aortic stenosis, heart failure and frailty, within the aging process in older patient candidates for interventional treatment of CAS with heart failure and a preserved ejection fraction (HFpEF). The two vertical dotted lines identify an ideal time for an appropriate interventional treatment in older patients.

We observed that the natural histories of aortic stenosis, heart failure, frailty in older patient candidates for interventional treatment of CAS, show a similar trend over time (Figure 1).

The first stage of the curves, which affects young/adult age, are flat, represents the phase of clinical asymptomaticity, and this implies a resilient condition thanks to the compensation mechanisms for the progression of aortic sclerosis, heart myocardial damage and the early phase of systemic aging characterized by robustness and phenotypic compensation of the biological aging. An adequate lifestyle and pharmacological control of CV risk factors (hypertension, diabetes, dyslipidemia, obesity) determine the duration of this phase.

In the more advanced stage of chronological age, the three curves may always be flat: calcific aortic stenosis progression becomes moderate/severe associated with cardiovascular and non-cardiovascular comorbidities; this presents a compensatory myocardial fibrosis with hypertrophy and heart failure with preserved (or not systolic) function; and a phenotypic compensation of systemic aging with reduced resilience and initial onset of frailty (prefrailty/early frailty). However, in this phase, older patients are still sensitive to external medications or effective interventional treatment.

During this phase, the concomitance of comorbidities levels is determinant and can mask the classic symptoms as dyspnea on exertion or angina. However, there is an external acute stressful event that worsens the prognosis by anticipating the change in the trajectory. For example, an acute heart failure event, heart attack, an acute decompensation [19] or arrhythmia with hospitalization, in addition to clinical worsening, accelerates the systemic aging mechanism, increasing a rapid progression of frailty and disability degree. In older patients with severe aortic stenosis, an AHF event with hospitalization modifies, at the same time, the trajectory of the curves towards progressive worsening of outcomes and mortality [11]. We imagined two vertical lines that cross the curves to define an ideal temporal window where an early interventional treatment of the valve leads to an effective result with better prognosis, just before the trajectories from flat become descending expressing a prognostic clinical change into the graph of chronological age (Figure 1). It is well evident that on the right of the temporal interval lines, there are both advanced and irreversible clinical/functional conditions of older patients with severe CAS and a preserved ejection fraction phenotype (HFpEF) that justifies the failure and futility of valve interventional treatment (SAVR/TAVR).

Therefore, it is possible to deduce that elderly patients (>75 years) with CAS require an accurate clinical/geriatric monitoring at shorter intervals to obtain benefit from an early interventional treatment at the mild/severe AS asymptomatic degree, corresponding chronologically to the left of the dividing line, certainly before the irreversible cascade trajectories.

A multidisciplinary integrated evaluation, during clinical surveillance of aortic stenosis, is crucial for an appropriate tailored decision making and for choosing the best time for effective interventional treatment of CAS through an overall monitoring of clinical signs, surgical, laboratory and imaging criteria integrated with scores of comorbidities, disability, physical frailty, nutritional and metabolic state, cognitive level, family and social conditions [30]. For patients with asymptomatic severe aortic stenosis and preserved left ventricular ejection fraction, current guidelines recommend routine clinical surveillance every 6 to 12 months. Moreover, more recent data of multicentric randomized clinical study highlight that among patients (mean age 75, 8 years) with asymptomatic severe aortic stenosis, a strategy of early TAVR was superior to clinical surveillance in reducing the incidence of death, stroke, or unplanned hospitalization for cardiovascular causes [29]. On the other hand, in patients with symptomatic and severity spectrum of AS undergoing AVR, the baseline cardiac damage has important prognostic implications and could be considered for timing and selection of therapy in patients with moderate or severe AS to determine the need for earlier AVR or adjunctive pharmacotherapy to prevent irreversible cardiac damage and to improve the long-term prognosis [6,9,12].

*Main points*

- *It is useful to establish the best timing for an effective interventional treatment of the CAS in relation to the trends of the progression of the valvular disease, myocardial damage and biological age.*
- *The natural histories of aortic stenosis, heart failure, and frailty in older patient candidates for interventional treatment of CAS show a similar trend over time.*
- *A multidisciplinary integrated evaluation, during clinical surveillance of aortic stenosis, is crucial for appropriate tailored decision making and for choosing the best time for effective interventional treatment, also for asymptomatic severe aortic stenosis.*

## 4. Severe Asymptomatic Aortic Stenosis: Early Intervention or Watchful Waiting

The timely management of asymptomatic severe AS is an important clinical dilemma for elderly complex patients. Four multicentric randomized controlled trials (RCTs) on asymptomatic severe AS have challenged traditional treatment paradigms considering a more individualized strategy for patients with high risk. The early TAVR trial among patients with asymptomatic severe AS reported that a strategy of early TAVR resulted superior to clinical surveillance in reducing the incidence of death, stroke, or unplanned hospitalization for cardiovascular cause [31]. The EVoLVeD study, that included asymptomatic patients with severe AS and myocardial fibrosis treated for early valve intervention with TAVR/SAVR or guideline-directed conservative management, had no demonstrable effect on all-cause death or unplanned aortic stenosis-related hospitalization [32]. The AVATAR trial showed that early surgery reduces a primary composite of all-cause death, acute myocardial infarction, stroke, or unplanned hospitalization for heart failure compared with conservative treatment, supporting early SAVR once AS becomes severe, regardless of symptoms [33]. RECOVERY, a multicenter randomized trial of asymptomatic patients with very severe aortic stenosis, highlighted that the incidence of the composite of operative mortality or death from cardiovascular causes during the follow-up period was significantly lower among those who underwent early aortic valve replacement surgery than among those who received a conservative care [34]. These studies showed no operative death, reflecting a good experience of operators; however, the cohort had a high proportion of patients with bicuspid aortic stenosis, which may justify early interventional treatment. Further, these results are not applicable to TAVR or to elderly patients. A pooled meta-analysis of the four randomized controlled trials (RCTs) has been performed and has been confirmed that early AVR in asymptomatic patients with severe AS is associated with a significant reduction in all-cause mortality, cardiovascular mortality, and heart failure hospitalization compared to conservative management [35]. We think that the results from RCTs, having short follow-up durations and low event rates suggest that early AVR does not reduce mortality in the short term, necessitating longer follow-up periods to reveal possible differences in mortality. Furthermore, in all the trials, the average mean age of patients is low, so it is probably associated with low comorbidity levels. However, they lack a multidimensional geriatric assessment that can establish the degree of comorbidity, physical frailty level, nutritional and cognitive status and mood tone, in order to also establish a prognosis on quality of life in both populations. Further, the findings support a reconsideration of new guidelines recommendations, for treatment of asymptomatic severe AS; but the cornerstone remains a multidisciplinary approach in decision making for appropriate treatment strategy in the risk–benefit balance, especially in severe asymptomatic degenerative AS, which represents the most increasing valve disease associated with aging of population.

## 5. Integrated Use of Biomarkers as Indices for Risk Stratification in Severe Asymptomatic CAS Can Guide the Timing of Interventional Treatment

### 5.1. Blood Biomarkers

The role of peripheral blood biomarkers in risk-stratifying asymptomatic patients with severe AS gives a simple, easy and accessible prognostic tool. These parameters could monitor aortic stenosis progression, LV myocardial damage, and remodeling phenotype, but also systemic low-grade inflammation, malnutrition, biological age and or frailty degree. They could serve as an auxiliary tool for risk stratification and optimization of the timing of intervention.

#### 5.1.1. Lipoprotein a [Lp(a)]

Among the biomarkers studied, lipoprotein(a) (Lp(a)) holds promise for risk stratification. Elevated Lp(a) levels are often associated with more rapid aortic stenosis progression [36] and recent Mendelian randomization observational studies have found elevated lipoprotein(a) as a potential pathogenic factor in AS progression, and lipid-lowering agents, as well as PCSK9 inhibitors, could be useful for preventive benefits on the calcification [37].

#### 5.1.2. Fetuin-A

Serum levels of the calcification inhibitor Fetuin-A have been associated with the progression of aortic valve calcification and major adverse cardiovascular events (MACEs), independently of renal function [38].

#### 5.1.3. Brain Natriuretic Peptide, NTproBNP, High-Sensitivity Cardiac Troponin T (hs-cTnT)

Among circulating biomarkers, B-type natriuretic peptide (BNP) is considered for the management of AS, recommending AVR for patients with asymptomatic severe AS when they have markedly elevated BNP. In fact, elevated BNP levels in asymptomatic patients with severe AS and preserved left ventricular function resulted in predicting an increased risk of adverse events with respect to those with lower BNP levels. However, BNP levels may also be associated with aging and comorbidities such as renal failure [39]. Stein EJ et al. reported that elevations of circulant cardiac biomarkers cTnT and NT-pro BNP are more common in left ventricular hypertrophy and are associated with maladaptive remodeling, symptom onset, and worse outcomes after TAVR [40]. More recently, the early TAVR study highlights that higher NT-proBNrP and hs-cTnT levels were significantly associated with higher event rates in patients with asymptomatic severe high-gradient aortic stenosis. Conversely, the relative benefit of an early TAVR strategy was consistent regardless of baseline biomarker levels and tended to be more pronounced in patients with the lowest biomarker levels [41].

#### 5.1.4. Systemic Inflammatory and Tissue Remodeling Biomarkers

Emerging biomarkers such as matrix metalloproteinases, monocytes, and metabolites show promise, but their specific roles in aortic stenosis pathophysiology remain less clear.

These data, as suggested by the authors, highlight a limited value for single measurements of these biomarkers to guide the timing of TAVR in asymptomatic patients, because they were associated with older age, worse functional status, worse renal function and the burden of multiorgan structural disfunction; therefore, it would be important to relate them to the level of comorbidity of elderly patients.

### 5.2. Imaging Biomarkers

Echocardiography remains a crucial diagnostic tool for the hemodynamic and anatomical assessment of aortic stenosis. Transthoracic echocardiography has been a well-established diagnostic modality for assessing the severity of AS according to current guidelines [7,8], but new structural and functional parameters of left ventricular and left atrial remodeling are proving useful in AS disease progression before evident symptoms appear.

Left ventricular global longitudinal strain (LVGLS) has emerged as a more sensitive measure of LV function and is able to identify patients with impaired systolic function despite a preserved LVEF. LVGLS is associated with markers of adverse LV remodeling, including myocardial fibrosis and increased hypertrophy. Several studies have demonstrated an association between impaired global longitudinal strain (GLS) and increased mortality in patients with AS, including among those with an LVEF > 50%. The presence of impaired GLS worsens the outcomes for adverse cardiovascular events in asymptomatic patients with AS regardless of the LVEF or AS severity or mean aortic valve pressure gradient [42]. These data highlight the importance of impaired LVGLS into risk algorithms in asymptomatic AS for a clinical management strategy and optimal time for interventional treatment.

Peak Atrial Longitudinal Strain (PALS). The left atrium is actually considered for emodinamic heart function; the new emerging role of LA strains may be useful for risk-stratification in patients with severe asymptomatic AS. In particular, patients with reduced PALS had significantly worse outcomes compared to those with normal values. PALS could be a potential marker of subclinical damage, leading to better risk stratification and earlier treatment [43].

Cardiac magnetic resonance (CMR). The actual interest within the natural history of AS focuses on the transition from compensatory LV hypertrophy to LV dysfunction and the biomarkers associated with myocardial wall stress, fibrosis, and myocyte death. CMR gives a non-invasive imaging parameters for optimizing the timing of aortic valve replacement and provide insight into novel biomarkers for possible prognostic use in AS. Asymptomatic patients with moderate-to-severe AS have demonstrated progression in the adverse cardiac remodeling within 12 months, with a significant increase in focal myocardial fibrosis [44]. Regarding myocardial fibrosis, it is well established that CMR, and specifically the LGE tool, can detect areas with a fibrotic pattern attributed to specific AS and in particular, by the combination of LGE and extracellular volume (ECV) could identify worse adverse LV remodeling, altered biochemical/histological parameters and consecutively the best stratification of AS patients according to the response of the myocardial collagen matrix [45]. Midwall myocardial fibrosis and ECV are in fact confirmed as robust negative prognostic indicators in symptomatic patients with severe AS, with incremental value for the development of cardiovascular events [46]. These data suggest focusing on CMR role in asymptomatic patients, on whether the timing of AVR should be guided by the initial development of midwall fibrosis to prevent further fibrosis progression and improve long-term clinical outcomes. However, further data from randomized clinical trials are necessary to define their utility in daily practice, detecting midwall fibrosis as a marker of early left ventricular decompensation.

### 5.3. Biomarkers of Aging

Aging is the main risk factor for cardiovascular disease. Calcific Aortic Valve Stenosis in the elderly is expression of structural cardiac disease, an intricate and multifaceted process with considerable impact on tissue cellular, and functional changes associated with cardiac ageing and heart failure with a preserved ejection fraction. Blood and imaging biomarkers of strain and fibrosis are hallmarks of both heart and systemic aging trajectory expression of biological and phenotypical aging [47]. However, while functional markers of systemic aging such as malnutrition, sarcopenia, high level of comorbidity and frailty have been shown to be important negative prognostic predictors in elderly patients undergoing SAVR/TAVR of severe symptomatic AS, we currently have no prospective randomized studies comparing interventional treatment versus clinical surveillance in elderly patients with asymptomatic severe AS using their hallmark by blood biomarkers or clinical scores [4,5,30].

## 6. New Perspective Features

Calcific aortic stenosis in the elderly is not only a focal valve disease, but a very complex clinical picture with different risk factors and etiologies, differing underlying pathophysiology, large phenotypic heterogeneity in a context of subjective biological aging.

Chronic cardiometabolic inflammation and oxidative stress are pivotal in the progression of CAS, driving valvular calcification, myocardial fibrosis but also systemic aging trajectory.

Future objectives must be directed to understand the biomolecular and physiopathological mechanisms to slow down these processes by pharmacological treatment before and after interventional treatment of CAS. Recently, SGLT2 inhibitors represent a novel pharmacological possibility to modify the natural history of CAS from the early asymptomatic stage to the post-interventional setting. SGLT2 inhibition, in fact, attenuates maladaptive remodeling through modulation of TGF-β, NF-κB, NLRP3 inflammasome, and oxidative stress signaling, enhancing mitochondrial energetics and endothelial function. Moreover, SGLT2 inhibitors improve heart failure outcomes following valve replacement and may slow AS progression, suggesting the possibility to delineate, in the future, the therapeutic rationale for SGLT2 inhibition in AS [48]. Ongoing and future trials are warranted to define optimal patient selection, timing, and biomarkers for response to SGLT2 inhibitor therapy in this increasingly high-risk population. Also elevated lipoprotein(a), considered as lipidic pathogenic factor in AS progression, could be useful for preventive benefits on the calcification by PCSK9 inhibitors [37].

Therefore, in future precision medicine, each patient might have the tailored appropriate interventional or pharmacological treatment of AS also considering the hallmarks of the aging process by use of artificial intelligence and omics (genomics, proteomics, metabolomics and transcriptomics), aiming for healthy aging.

Currently, TAVR has become the preferred treatment option in patients with symptomatic severe aortic stenosis selected by appropriate multidimensional assessment to avoid futility and potential harm and to provide the best individual clinical decision making. Accurate patient selection, careful procedural planning, (access route, technique, type and size of the valve) and adequate postoperative care (rehabilitation measures and drug therapy) contribute to reducing the mortality risk of TAVR. High levels of comorbidities, severe frailty as well as the presence of some specific geriatric conditions such as advanced dementia, sarcopenia/cachexia, disability and poor mobility should strongly discourage the procedure. Conversely, aortic valve intervention in paucisymptomatic patients, during the pre-frailty stage, with low comorbidity and HFpEF phenotype, has shown good short- and long-term prognosis.

**Figure 2:**
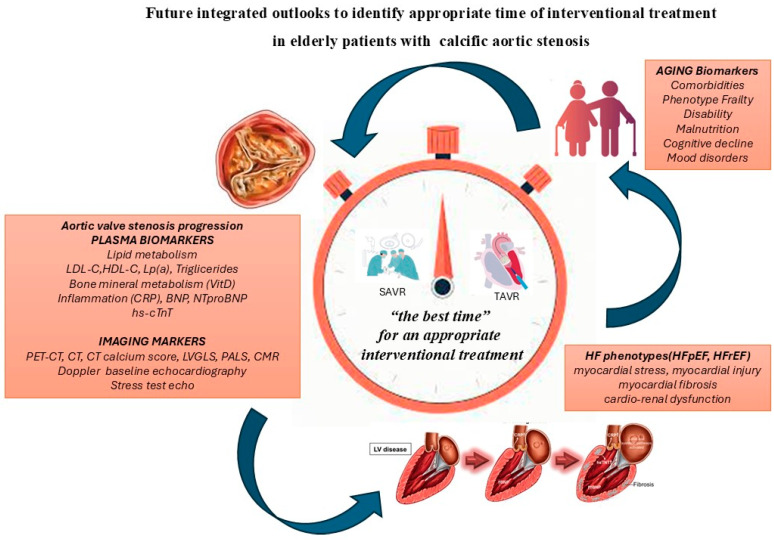
A strategy of multidimensional evaluation during clinical surveillance in elderly patients with calcific aortic stenosis to identify the appropriate time for interventional treatment.

So, in future personalized medicine, each elderly patient might have “the subjective best time” for an appropriate interventional treatment of AS also considering, over chronological age, the three concomitant and similar trajectories of aortic valve disease progression, heart failure phenotype and biological age. Integrating variables—including clinical and surgical features, HF biomarkers, imaging, genotypes, metabolomics, and proteomics —can identify the best time for an appropriate interventional strategy of CAS in each older patient (Figure 2).

In a global context that predicts an increase in aging in the coming decades, artificial intelligence could be of great help in measuring the complexity of each patient to select a personalized and effective treatment.

## 7. Conclusions

Calcific aortic stenosis in elderly patients is not only a focal valve disease, but a very complex clinical picture. CAS is a structural degenerative disease of the valve, and the heart is part of the systemic aging process.

Many randomized studies are aimed at early interventional treatment of AS in the asymptomatic phase, giving good short- and long-term prognostic prospects.

Further, the ability to monitor during surgical timing the trajectories of these three natural histories of aortic valve, myocardial phenotypic damage and systemic aging through integrated clinical, geriatric, laboratory and imaging biomarkers, can currently help us to define the ideal interval time for appropriate interventional treatment of the valve before the onset of cardiac and geriatric clinical complications.
